# A Systematic Review of Fatalities Related to Acute Ingestion of Salt. A Need for Warning Labels?

**DOI:** 10.3390/nu9070648

**Published:** 2017-06-23

**Authors:** Norm R. C. Campbell, Emma J. Train

**Affiliations:** 1 Department of Medicine, Physiology and Pharmacology and Community Health Sciences, O’Brien Institute for Public Health and Libin Cardiovascular Institute of Alberta, University of Calgary, Calgary, AB T2N 4Z6, Canada; 2 The School of Public Policy, University of Calgary, Calgary, AB T2N 4Z6, Canada; emmajtrain@gmail.com

**Keywords:** salt, sodium, overdose, warning labels, hypertension, hypernatremia

## Abstract

There are sporadic cases of fatalities from acutely eating salt. Yet, on social media, there are “challenges to” and examples of children and some adults acutely eating salt, and recently a charity advocated eating small amounts of salt to empathize with Syrian refugees. We performed a systematic review of fatalities from ingesting salt to assess if relatively moderate doses of salt could be fatal. In 27 reports, there were 35 fatalities documented (19 in adults and 16 in children). The lethal dose was estimated to be less than 10 g of sodium (<5 teaspoons of salt) in two children, and less than 25 g sodium in four adults (<4 tablespoons of salt). The frequency of fatal ingestion of salt is not able to be discerned from our review. If investigation of the causes of hypernatremia in hospital records indicates salt overdose is relatively common, consideration could be given to placing warning labels on salt containers and shakers. Such warning labels can have the added advantage of reducing dietary salt consumption.

## 1. Introduction

Dietary risks are estimated to be the leading risk for death and disability globally [1]. Among the dietary risks, high intake of dietary sodium is the leading risk, being attributed to over 4 million deaths and 83 million disability-adjusted life years (DALYs) in 2015 [1]. The deaths and disability are considered to be largely related to hypertension and dietary salt intake promoting gastric cancer [1].

Much less attention has been given to the acute toxic effects of dietary salt. Isolated cases of fatalities caused by the acute ingestion of table salt have been reported [2]. These isolated reports were concerning when a Canadian campaign was initiated in August of 2016 to encourage people to eat small quantities of table salt to empathize with the suffering of refugees in Syria [3]. The #Salt4Syria marketing campaign website shows children eating salt out of spoons and pictures of an overflowing spoonful of salt. The campaign encourages children to record and share their salt consumption videos and nominate others to do the same. Further, a brief investigation of social media indicates several children and adults are acutely ingesting salt and challenging others to do so (a google video search of “eat salt challenge” resulted in 313,000 uploaded videos, 20 April 2017). A systematic review of fatalities occurring from acute ingestion of salt was conducted, in part, to assess the potential risk of advocating the acute ingestion of salt. The intent was to assess whether relatively modest ingestion of salt might cause fatalities.

## 2. Materials and Methods

We systematically searched for all studies that reported fatalities from ingesting salt (sodium chloride). The search strategy included Medline, Embase, Cochrane Register of Control Trials, and the Cochrane Database of Systematic Reviews using the terms from Table 1 on 23 January 2017 with no time restrictions. NRCC searched all the titles and abstracts identified by the searches, and articles that were identified as potentially eligible were obtained for full review. The articles were reviewed by NRCC. The reference lists of the included studies and reviews were hand-searched for additional studies. Letters to editors and abstracts were included.

Articles were included if there was oral ingestion of salt (sodium chloride) that resulted in a fatal outcome. Fatalities related to gastric lavage with salt were included but fatalities related to intravenous and rectal administration of salt and to absorption of salt through the skin (burn treatment) were excluded. There were no exclusion criteria based on language.

Data from manuscripts was extracted by EJT and NRCC, including the first author, year of publication, country where the report originated, location, and the patients’ age, gender, weight (kg), category of ingestion (e.g., accidental by patient, intentional by patient (suicide), therapeutic adverse outcome from lavage), estimated dose of salt/sodium ingested, presence of known chronic disease, presence of known prior renal dysfunction, co-ingestion of other toxins, co-ingestion of drugs that impact renal excretion of sodium (e.g., diuretic), mental illness, acute illness, and highest recorded serum sodium level. The fatalities were categorized by age (<5, 5–10, >10 to 18 and >18 years). Where the amount of salt ingested was provided in a range, this was recorded.

Salt and sodium ingestion units were converted to sodium in grams using the conversions: 1 mmol sodium = 1 mEq sodium = 23 mg sodium, and 1 g sodium = 2.54 g salt (NaCl). Where salt ingestion was estimated by table, dessert, or teaspoons, the ingested salt was estimated to be 17.06 g (sodium 6.8 g), 11.4 g (sodium 4.5 g), and 5.7 g (sodium 2.28 g), respectively. Where it was indicated that the spoon was overfilled or “large”, 50% more salt was estimated to have been ingested. When estimates were provided both as a weight (e.g., 60 g salt) and a spoon measurement (2 tablespoons), both are indicated as a range. When sodium bicarbonate was co-administered with sodium chloride, the total sodium dose ingested was estimated.

## 3. Results

**Figure 1:**
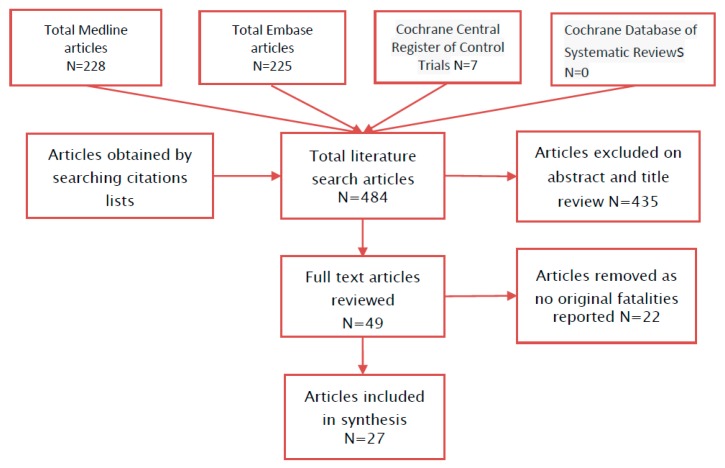
Flow diagram.

The literature search yielded 460 articles (Figure 1) of which 25 articles were selected for full-text review following a review of the title and abstract. Exclusions included 13 articles that did not report fatality from salt ingestion, and one article was a review without original cases reported. Eleven of the included articles reported one or more fatalities from acute ingestion of salt. Review of abstract and titles of the citations in the selected articles obtained in the literature search yielded an additional 24 articles for full text review. Of the 24, 17 contained reports of fatalities from ingesting salt. Thus, 27 manuscripts reporting fatality from ingesting dietary salt were obtained. There were 35 fatalities reported, 19 in adults [2,4,5,6,7,8,9,10,11,12,13,14,15,16,17,18,19] and 16 in children [6,20,21,22,23,24,25,26,27,28,29]. The fatalities were categorized by age (Table 2 and Table 3). There were no fatalities reported in the age category 10–18 years. Nearly all the reported cases had multiple missing values in the data extraction table with, for example, few reporting weights. 

Adults (age >18): There were 19 fatalities reported in 16 females and 3 males (average age 40.6, standard deviation (s.d.) 17.9 years, range 19–83) (Table 2). In twelve fatalities, the salt was administered as an emetic, in four the salt was administered as part of an exorcism ritual, in three ingestion was inadvertently mistaking salt for sugar, and in one the reason for ingestion was unknown. The average lower and average higher estimated doses of sodium ingested was 60 and 118 g, respectively. The ingested doses in individuals were estimated to range from 6 g to 400 g. In four fatalities, the estimated sodium dose ingested was under 25 g.

In two of the fatalities, sodium bicarbonate was co-ingested with salt (sodium chloride). In the cases where salt was given as an emetic, it was for overdoses with several of the overdoses reported as minor. Although many of the fatalities had histories of prior psychiatric disease, few had chronic medical disease and none were previously known to have renal impairment. The average maximum blood/serum sodium level recorded was 205.51 (s.d. 28.19) mmol/L, with the highest being 255 mmol/L and the lowest 151 mmol/L.

Adolescents (age 10–18 years): There were no reported fatal cases in this age range.

Children (age 5–10 years): There was one reported fatality (female age 5) (Table 3). The dose of sodium ingested was estimated to be a minimum of five teaspoons of table salt given by the parents (approximately 11.4 grams of sodium). The patient had a record of poor growth but was otherwise healthy and had a serum sodium level of 220 mmol/L after ingestion.

Children (age <5 years): There were 15 reported fatalities overall and 13 fatalities that reported some individual details (Table 3). The ages ranged from 1 day to 4 years of age. In eight of the fatalities, salt was mistaken for sugar, with six occurring in a single incident at a hospital. The salt was reported to be administered by parents in many of the remaining fatalities with intent to injure, and as an emetic in four cases. In one fatality, there was a mistake made in the amount of salt added in making a rehydration formula. Most of the doses of sodium ingested were not known, with reported estimates ranging from less than 7 g to 13 g. Similarly, most serum sodium levels were not reported, but reported levels ranged from 178 to 245 mmol/L.

## 4. Discussion

Relatively modest doses of sodium have been reported to cause fatality. In two children, the lethal dose was estimated to be less than 10 g of sodium (less than five teaspoons of salt) and the lethal dose was estimated to be less than 25 g sodium in four adults (less than four tablespoons of salt). The mechanism of salt ingestion causing death is believed to be related to hypernatremia with the serum sodium levels in reported fatalities ranging from 175 to 255 mmol/L. Ingestion of as little as two tablespoons of salt has been reported to increase serum sodium levels by as much as 30 mmol/L with the potential to cause severe irreversible neurological damage [7,9]. Many of the fatalities were related to the administration of salt therapeutically as an emetic agent, where toxicity may have been, in part, related to co-ingested drugs. Nevertheless, several deaths occurred by accidental ingestion of salt, most where salt was mistaken for sugar without any other co-ingested toxins. While this review found that modest amounts of sodium have been reported to be associated with death, the review design and available information do not allow any estimates of the usual amount of ingested sodium that might be lethal (e.g., lethal dose 50 (LD50); the dose that is lethal in 50% of exposed animals). The media LD50 in rats is 3 g/kg for ingestion over 4 h [30]. Further, the reported deaths and serious illness from using salt as an emetic agent resulted in strong recommendations to no longer use salt for that indication [10]. Historically it has been reported that wealthy Chinese committed suicide by ingesting large amounts of salt [6].

The lower amounts of sodium ingested that were associated with death in this systematic review are only four-fold higher than daily intake levels consumed by the average person in Beijing and less than twice as high as the upper range of daily consumption by individual Chinese [31,32]. Ingestion of sodium in forms that are rapidly consumed and absorbed (e.g., dissolved in water) may acutely overwhelm the ability to excrete sodium resulting in rapidly increasing extracellular (and specifically serum) sodium levels. Humans evolved on much lower amounts of sodium than are currently ingested (0.1 to 1 g sodium/day), and hence we may not have developed the capacity to rapidly excrete large amounts of sodium that are acutely ingested [33,34]. Excretion is by the kidneys, sweating, and gastrointestinal tract [33]. About 90% of ingested sodium is excreted by the kidneys [33]; hence, it is reasonable to hypothesize that individuals with less capacity to excrete sodium (i.e., renal impairment) might be particularly susceptible to toxic effects ingested sodium. In the systematic review, there were no cases where renal impairment was identified before the overdose, although several persons were noted to have a modest elevation in serum creatinine (≤140 µmol/L) during the acute illness following sodium ingestion [2,4,5,9]. A wide variation in ability to excrete sodium has been observed in studies examining genetic variation in the blood pressure increasing effects of dietary sodium [35]. Hence, through genetic variation in renal excretion of sodium, some “salt-sensitive” individuals may be predisposed to, and some “salt-resistant” individuals protected from, the adverse effects of acute sodium ingestion. The sodium content in food is likely to be lower and more slowly absorbed than the ingestion of salt itself or in solutions, hence these findings are not likely to inform the discussion on salt as generally regarded as safe (GRAS) additives which has been ongoing in the United States [36]. Nevertheless, very severe hypernatremia has been reported from drinking food condiments high in sodium (e.g., soya sauce) [37].

There are several weaknesses to this systematic review. For many if not most of the fatalities, the ingested dose of salt could only be estimated based on incomplete information. In particular, the estimated dose of sodium of 6 g causing a fatality in an adult seems implausible. We have also noted that most reports gave incomplete information on the patients, including such important information as weight that could have allowed the doses of salt ingested to be expressed per kg of body weight. The review does not provide useful information on how frequent fatal salt ingestion occurs. Further, the levels of hypernatremia reported are likely underestimates of the true peak blood sodium levels, as in several of the fatalities, blood levels of sodium were not assessed at presentation nor were they closely monitored. Lastly, many of the manuscripts were found by hand-searching reference lists as opposed to the primary literature searches. Factors which may have reduced the sensitivity of the literature searches may include the older age of many publications, the relatively low prominence of several of the journals, and that several of the articles were “letters” to editors rather than full research publications.

If severe hypernatremia is relatively common with doses of salt ingestion as low as reported in this review, it may speak to the need to place warning labels on salt shakers and containers. Several of the reported cases came from a review of single-centre case reviews, suggesting that a systematic examination of patients presenting with hypernatremia may find a higher frequency of fatal sodium overdoses than this systematic review of published literature indicates [6,22,26]. Warning labels could increase the awareness of the acute dangers of salt and reduce the number of people ingesting salt as part of a challenge or ritual and may discourage organizations from using salt ingestion in awareness campaigns. A search on social media finds many recent examples of mostly children and some adults challenging themselves or others to eat amounts of salt that are in the range of the fatalities reported in this systematic review (google search “eat salt challenge”, 10 April 2017). An added benefit of warning labels on salt containers relates to evidence that it may reduce salt intake [38,39,40].

## 5. Conclusions

This systematic review finds doses of salt that have been reported to cause fatalities are not exceptionally high and accidently replacing sugar with salt can cause fatalities. The prevalence of salt overdose is uncertain, but if it is relatively common as suggested on social media, warning labels on salt and high-salt products (e.g., soya sauce) could be one mechanism considered to reduce the use of salt in rituals and salt challenges.

## Figures and Tables

**Table 1:** Literature search terms to identify potential fatalities related to salt ingestion.

1.	((toxic* or intoxic* or overdos*) adj5 salt).mp
2.	((toxic* or intoxic* or overdos*) adj5 Na).mp
3.	((toxic* or intoxic* or overdos*) adj5 sodium).mp
4.	((salt* or Na or sodium) adj5 overdos*).mp
5.	hypernatremia.mp
6.	hypernatremia/
7.	1 or 2 or 3 or 4 or 5 or 6
8.	((dietar* or intake* or food* or consumption or consume* or ingest*) adj5 sodium).mp
9.	((dietar* or intake* or food* or consumption or consume* or ingest*) adj5 salt).mp
10.	((dietar* or intake* or food* or consumption or consume* or ingest*) adj5 Na).mp
11.	8 or 9 or 10
12.	7 and 11
13.	Limit 12 to humans

**Table 2:** Fatal ingestion of salt in adults.

First Author Year of Publication	Age (Years)	Gender	Explanation of Overdose	Estimated Dose Ingested Sodium (g) *	Highest Reported Blood Level of Sodium (mmol/L) **	Co-Ingestion of Other Potential Toxins	Chronic Illness
Engjom 2008 [4]	83	Female	Mistaken for sugar	13.6–20.4 g	223	None stated	Hypertension, dementia
Raya 1992 [5]	36	Female	Exorcism ritual	273 g	246	Sodium bicarbonate *	None stated
Turk 2005 [6]	34	Female	Health care professional administered emetic	80 g	196	None stated	“Psychomotor retardation”
	69	Male	Health care professional administered emetic	39.4–81.6 g	175	Single table of unprescribed “neuroleptic”	Schizophrenia
Moder 1990 [2]	41	Male	Mistaken for sugar	27.1–34.8 g	209	None stated	Downs Syndrome, lymphoma, hepatitis B, seizures
Johnston 1977 [7]	45	Female	Mistaken for sugar	30.6–40.8 g	190	None stated	Prader-Willi Syndrome, hypertension obesity, impaired glucose tolerance
Bacarreza 2008 [8]	33	Female	Not known	50 g	203	None stated	Alcohol abuse
Ofran 2004 [9]	20	Female	Exorcism ritual	<400 g	255	None stated	Depression
Robertson 1971 [10]	23	Female	Emetic	Not stated	214	Chlordiazepoxide overdose	None stated
Hey 1982 [11]	56	Female	Emetic	27.2–47.2 g	214	“trivial” overdose	Not stated
Hedouin 1999 [12]	19	Female	Exorcism ritual	Not stated	153	None stated	Hydrocephalus, seizures
Bird 1974 [13]	35	Female	Emetic	20.4 g	200	“Overdose”	“Psychiatric patient”
Ward 1963 [14]	74	Male	Emetic	13.6–24.4 g	174	Accidental overdose of imipramine and perphenazine	“Mild depression”
Gresham 1982 [15]	48	Female	Emetic	6.8–10.2 g	166	“Extra dose” of chlorpromazine	Past leucotomy, depression
Laurence 1969 [16]	35	Female	Emetic	69 g	184	Overdose thioridazine	None stated
Goodbody 1975 [17]	35	Female	Emetic and lavage	>17.7 g	226 ***	“Minor” overdose sodium amytal	None stated
	44	Female	Emetic	Not stated	151	Overdose sodium amytal	None stated
Winter 1974 [18]	21	Female	Emetic and lavage	118–236 g	227	Amitriptyline, imipramine, chlorpromazine, diazepam and nitrazepam overdose	Psychiatric disease
Roberts 1974 [19]	26	Female	Emetic	27–60 g	172	Salicylate overdose	Depression

* The sodium in g from the sodium bicarbonate or lavage is included in the estimate of ingested sodium; ** reports did not indicate if blood or serum levels were provided; *** post-mortem value, 210 mmol/L was recorded pre-mortem.

**Table 3:** Fatal ingestion of salt in children aged 10 years and under.

First Author, Year of Publication	Age (Years) (Months) (Weeks) (Days)	Gender	Explanation of Overdose	Estimated Dose Ingested Sodium (g) *	Highest Reported Serum Level of Sodium (mmol/L) **	Co-Ingestion of Other Potential Toxins	Chronic Illness
Dockery 1992 [21]	5 years	Female	Parental administration	11.4 g	220	None stated	“Poor growth”
Turk 2005 [6]	4 years	Female	Emesis	Not stated	245	None stated	Low body weight
Martos Sanchez 2000 [22]	20 months	Female	Mistaken for sugar	9.12 g	195	None stated	None stated
	7 months	Female	Accidental	5.03 g	178	None stated	None stated
Scott 1947 [23]	2 years	Male	Mistaken for sugar	<7 g	Not stated	None stated	Gastrointestinal strictures
Barer 1973 [24]	3 years	Male	Emetic and lavage	Not stated	188	Aspirin overdose	None stated
Streat 1982 [25]	2 years	Female	Emetic	Not stated	204	Pheniramine overdose	None stated
Finberg 1963 [26]	7 days	Male	Mistaken for sugar	Not stated	Not stated	None stated	Prematurity
	2 months	Female	Mistaken for sugar	Not stated	Not stated	None stated	Congenital neuroblastoma
	3 weeks	Male	Mistaken for sugar	Not stated	Not stated	None stated	None stated
	5 days	Female	Mistaken for sugar	Not stated	Not stated	None stated	None stated
	2 days	Female	Mistaken for sugar	Not stated	Not stated	None stated	None stated
	1 day	Female	Mistaken for sugar	Not stated	244	None stated	None stated
Rogers 1976 [27]	1 year	Female	Parental administration	Not stated	200	None stated	Repeated abscesses
Meadow 1993 [28] ***	1.5–9 months	Half were female	Parental administration	Not stated	Not stated	Not stated	Not stated
Smith 1990 [29]	26 months	Not stated	Emetic for minor overdose	6.8–13.6 g	217	None stated	None stated

* The sodium in g from lavage is included in the estimate of ingested sodium where possible; ** reports did not indicate if blood or serum levels were provided; *** two fatalities were reported in a case series but individual data was not provided.
